# An integrative approach to identify novel miRNA-mRNA interaction networks in *LMNA-*cardiomyopathy

**DOI:** 10.1038/s41598-026-36439-9

**Published:** 2026-01-24

**Authors:** José Córdoba-Caballero, Fernando Bonet, Oscar Campuzano, Georgia Sarquella-Brugada, Ignacio Perez de Castro, Borja Vilaplana-Martí, Pedro Seoane-Zonjic, Alipio Mangas, Juan A. G. Ranea, Rocio Toro

**Affiliations:** 1 Biomedical Research and Innovation Institute of Cadiz (INiBICA), Research Unit, Puerta del Mar University Hospital, Cádiz, Spain; 2 Department of Molecular Biology and Biochemistry, University of Málaga, Malaga, Spain; 3 H12O-CNIO Haematological Malignancies Clinical Research Unit, Spanish National Cancer Research Centre, Madrid, Spain; 4 Medicine Department, Medical School of Cádiz, Cádiz University, 11003 Cádiz, Spain; 5 Medical Science Department, School of Medicine, University of Girona, Girona, Spain; 6 Institut d’Investigació Biomèdica de Girona (IDIBGI-CERCA), Salt, Spain; 7 Centro Investigación Biomédica en Red, Enfermedades Cardiovasculares (CIBERCV), Madrid, Spain; 8 Pediatric Arrhythmias, Inherited Cardiac Diseases and Sudden Death Unit, Cardiology Department, Sant Joan de Déu Hospital, Barcelona, Spain; 9 Arrítmies Pediàtriques, Cardiologia Genètica i Mort Sobtada, Malalties Cardiovasculars en el Desenvolupament, Institut de Recerca Sant Joan de Déu, Barcelona, Spain; 10 Instituto de Investigación de Enfermedades Raras , Instituto de Salud Carlos III, Madrid, Spain; 11 Servicio Cirugía Experimental, Instituto Aragonés Ciencias de la Salud (IACS), Zaragoza, Spain; 12 Department of Molecular Biology and Biochemistry, University of Málaga, Malaga, Spain; 13 Internal Medicine Department, Puerta del Mar University Hospital, Cádiz, Spain; 14 Institute of Biomedical Research in Málaga (IBIMA Plataforma BIONAND), Malaga, Spain

**Keywords:** *LMNA-*related dilated cardiomyopathy, MicroRNA, mRNA, RNA-sequencing, miRNA-gene interaction, Biological pathways, Computational biology and bioinformatics, Systems biology, Cardiology, Molecular medicine

## Abstract

Dilated cardiomyopathy caused by variants in the *LMNA* gene leads to malignant arrhythmogenic events, faster phenotype progression and high risk of sudden cardiac death. The pathophysiological mechanisms triggering disease progression remains poorly understood. We investigated the mRNA and miRNA transcriptome in the myocardial tissue of 50-week-old *LMNA*^*R249W*^ mice developing dilated cardiomyopathy. We found 2148 genes and 53 miRNAs that were differentially expressed in *LMNA*^*R249W*^ hearts. Gene ontology and pathway enrichments showed that differentially expressed genes were enriched mainly for fatty acid metabolism, muscle contraction, cell adhesion and dilated cardiomyopathy pathways. The miRNA-mRNA interactions analysis identified 2197 miRNA-target pairs with an anti-correlation between differentially expressed genes and miRNAs. Gene ontology and pathway enrichments revealed that the most significant functions of miRNA targets are mainly related to heart development, cardiac muscle contraction, fatty acid β-oxidation, cell adhesion and calcium binding pathways, among others. Our study provides new insights into the molecular mechanisms that determine dilated cardiomyopathy due to pathogenic variants in the *LMNA* gene, and identified several target pairs that are of potential interest for further studies.

**Supplementary Information:**

The online version contains supplementary material available at 10.1038/s41598-026-36439-9.

## Introduction

Dilated cardiomyopathy (DCM) is a cardiac disorder that represents the major cause of heart failure and cardiac transplantation worldwide^1,2^. This disease is characterized by enlargement of the left ventricle and progressive deterioration of systolic function^3^. The natural history of DCM is determined by its heterogeneous etiology, due to both genetic and non-genetic causes^4^. Nowadays, between one-third to one-half of patients with idiopathic DCM have a genetic origin^5^. Pathogenic alterations in more than 30 genes have been linked to the genetic form of DCM. Among them, deleterious variants in the *LMNA* gene are responsible for approximately 10% of cases, mainly following a dominant pattern of inheritance^6–8^. The *LMNA* gene encodes nuclear lamin A and C, intermediate filament proteins that are critically important to the structural properties of the nucleus, as they provide nuclear shape and mechanical stability. Several studies have also supported its role in DNA replication, gene expression, chromatin organization, and cell cycle progression^9^. A significant number of patients diagnosed with DCM caused by pathogenic alterations in the *LMNA* gene (*LMNA*-DCM) display complications only in the cardiovascular system^10^. Furthermore, most reported *LMNA*-DCM patients exhibit more aggressive arrhythmogenic behavior, thromboembolisms with fatal outcomes, and faster phenotype progression compared to other forms of DCM^11^. In addition, the cardiac dysfunction is often preceded by the conduction system disease and/or arrhythmia, which is a major concern for the cardiologist^12^. Both incomplete penetrance and variable expressivity, influenced by external factors, difficult the risk stratification of *LMNA*-DCM patients. Current medical treatment for *LMNA*-DCM follows the standard heart failure management recommendations, including pharmaceutical and non-pharmaceutical therapies, with suboptimal results. Hence, understanding the underlying pathophysiological mechanism of this malignant entity is urgently needed to shed light on novel treatment approaches^13,14^.

MicroRNA (miRNA) are single-stranded non-coding RNA (ncRNA) of ~ 22 nucleotides that repress gene expression by binding to complementary sequences in the 3’ untranslated region (3’ UTR) of mRNAs to target them for degradation or translation suppression^15^. A single miRNA can target multiple genes and a single gene can be targeted by many miRNAs^16^. Numerous studies have demonstrated critical functions of miRNAs in the progression of various DCM etiologies^17^. However, the role of miRNAs in the onset, progression and outcome of *LMNA*-DCM remains to be deeply analyzed so far. Although prior RNA-sequencing (RNA-seq) studies have evidenced different gene expression profile in distinct *Lmna* mutant mouse lines as compared to wild-type, to date no miRNA sequencing studies on *Lmna* mutants have been conducted, with only a brief analysis being reported^18^. The aim of this study was to identify crucial gene-miRNA pairs to elucidate the mechanisms of miRNA regulation in *LMNA*-DCM progression. For this purpose, we used high-throughput mRNA and miRNA-seq of myocardial tissue from 50-week-old wild-type and a *Lmna* mutant mouse line, namely *LMNA*^*R249W*^, characterized by a unique DCM phenotype. Then, we performed a comprehensive analysis of the miRNA-mRNA interactome to identify important molecular mechanisms regulating the pathogenesis of *LMNA*-DCM.

## Materials and methods

### Mice

The murine model used in our study carries the widely reported pathogenic variant p.Arg249Trp (p.R249W, c.745 C > T, rs121912496) in the *LMNA* gene, the most prevalent in *LMNA*-related congenital muscular dystrophy (L-CMD) a type of laminopathy characterized by the development of dilated cardiomyopathy^19^. Our mouse model (*Lmna*^*R249W*^), which has been recently used to explore the potential of CRISPR technology for the treatment of L-CMD^20^, recapitulates many of the features of L-CMD and its detailed characterization will be published elsewhere. Cardiac function is severely affected in *Lmna*^*+/R249W*^ mice leading to a premature death. Echocardiographic analyses show that *Lmna*^*+/R249W*^ mice develop dilated cardiomyopathy characterized by an increase in the telesystolic and telediastolic diameters and volumes of the left ventricle, as well as a reduction in the ejection fraction and shortening fraction compared to controls. These changes increase with age and are always significative at 50 weeks of age. At the histological level, 50-week-old, *Lmna*^*+/R249W*^ mice are characterized by a marked fibrosis, a feature that it is not detected among the wild type counterparts. Here, we have analyzed both mRNA and miRNA expression profiles by RNA-seq in myocardial tissue of 50-week-old *Lmna*^*R249W*^ (*n* = 6, 3 males plus 3 females) and age-matched wild-typed type mice (*n* = 6, 3 males plus 3 females). Mice were housed at the specific pathogen-free barrier area of the Instituto de Salud Carlos III (ISCIII) (Madrid, Spain). Mice were observed daily and sacrificed when they showed overt signs of morbidity in accordance with the Guidelines for Humane Endpoints for Animals Used in Biomedical Research from the Council for International Organizations of Medical Sciences (CIOMS). All animal procedures were performed according to the procedures (PROEX 164 -18) approved by the ISCIII Ethics Committee for Research and Animal Welfare (CEIyBA) and are conformed to the ARRIVE guidelines as well as those from Directive 2010/63/EU of the European Parliament on the protection of animals used for scientific purposes. No anaesthetic was used in this work. The euthanasia was performed using a 30–70% per minute displacement of chamber air with compressed CO2. This study was approved by the Andalusian Biomedical Research Ethics committee (CEICA, reference number PI17/0023). The ethical research principles were fulfilled following the Helsinki Declaration and the Belmont report. The study also adhered to two legal provisions governing human research and the Spanish Organic Law 15/1999 for the Regulation of Automated Processing of Personal Data.

### RNA isolation and qRT-PCR

Hearts were mechanically homogenized and stored at -80 °C for approximately one year until total RNA was extracted and purified by using TRI Reagent (Sigma-Aldrich, St Louis, MO, USA) according to the manufacturer’s instructions followed by DNase treatment and purification using RNA clean and concentrator-5 kit (Zymo Research, Irvine, CA, USA). RNA was quantified using a Qubit RNA High-Sensitivity Assay kit in the Qubit^®^ 2.0 Fluorometer (Life Technologies, CA, USA). For miRNA expression analysis, 5 ng of RNA was reversed transcribed using miRCURY LNA RT Kit (Qiagen, Hilden, Germany) according to manufacturer´s instruction. qRT-PCR was performed on a CFX96 Real-Time PCR system (Bio-Rad) using the miRCURY LNA SYBR Green PCR Kit (Qiagen, Hilden, Germany), expression was normalized against U6 and 5s, and data was analyzed using the 2^−∆∆Ct^ algorithm.

### RNA sequencing

The quality and integrity of total RNA were controlled on the Agilent Technologies 2100 Bioanalyzer. All samples showed RNA Integrity Numbers (RIN) above 8.0, indicating excellent RNA quality suitable for sequencing. Standard-specific mRNA-seq libraries were generated using the NEBNext Ultra II Directional RNA Library Prep Kit for Illumina using the NEBNext Poly(A) mRNA Magnetic Isolation Module (New England Biolabs, Ipswich, MA). Single-end sequencing (75 bp, SE75) was performed on an Illumina NextSeq 500 High Output Platform, yielding an average of 70.7 M reads per sample. Standard miRNA libraries were generated using the NEXTFLEX small RNA-seq kit v3 (Perkin Elmer, Waltham, MA, USA), and single-end sequencing (75 bp, SE75) was performed in the same platform, with an average output of 22.1 M reads per sample. The original mRNA and miRNA sequencing data from mice at 50 weeks are available in the NCBI BioProject database under accession number PRJNA1232038.

### Gene and MiRNA expression analysis

RNA-seq and small RNA-seq samples were separately quantified using differential gene expression (DEG)_workflow^18^. RNA-seq samples are preprocessed with SeqtrimBB using the default template for transcriptomic data to remove sequencing artifacts, low-quality bases, and adapter contamination. Preprocessed reads are then aligned to the reference genome and quantified with STAR. The used reference genome is GRCm38, ensuring the use of the most stable assemblies and gene annotations available at the time of the analysis.

The mapping step generates count tables representing the number of reads assigned to each gene in each sample. These raw counts are normalized to Counts Per Million (CPM), which adjusts the raw read counts according to the sequencing depth of each library, thereby allowing gene expression levels to be compared across samples. Specifically, CPM represents the number of reads mapped to a gene per million total mapped reads in a sample, providing a standardized expression measure that accounts for variations in sequencing depth. Differential expression analysis was performed using ExpHunterSuite^21^, which executes edgeR, limma, NOISeq and DESeq2 (using default parameters) algorithms with the deg_hunter.R script. ExpHunter Suite applies a system to classify differentially expressed genes. Genes are considered prevalent DEGs (Differential Expressed Genes) if they are detected by at least as many packages as the user-defined threshold, while possible DEGs are those identified by at least one method but fewer than the threshold. In this work, DEGs are defined using a significance threshold of adjusted p-value < 0.05, absolute logFC ≥ 1 and these thresholds mut be meet for three of the four algorithms used in the analysis. Additionally, the Weighted Correlation Network Analysis (WGCNA) is applied to obtain gene co-expression modules along with the DEG analysis. Finally, the overrepresentation analysis of Gene Ontology (GO) terms and Kyoto Encyclopedia of Genes and Genomes (KEGG) and Reactome pathways in DEGs was analyzed using functional_hunter.R. KEGG’s freely accessible resources were used solely for data querying, and the graphics were designed at our center.

In the case of the miRNA-seq, preprocessed reads with SeqtrimBB were mapped to the mice reference genome using Bowtie. Then, preprocessed reads and combined mapping files were analyzed with miR-Deep2 using known miRNAs for mouse, previously downloaded from the miRBase database. Detected miRNAs in all samples were merged to produce a fasta file in which redundancy was removed by merging similar sequences using CD-HIT-EST. Preprocessed reads were mapped to the non-redundant predicted miRNA fasta using Bowtie2 with default options for single reads and were quantified using sam2counts. This resulted in a table of counts for all miRNAs across samples. This count table is analysed in the same manner that the gene expression counts applying the deg_hunter.R script. A Differential expressed miRNA (DEM) is identified when the four algorithms, edgeR, limma, NOISeq and DESeq2 calculates a Log 2-Fold Change equal or greater to 1 and an adjusted p-value of 0.05.

### miRNA-gene target network analysis

The lists of DEG and DEM from the expression analysis and the coexpression modules identified for genes and miRNAs obtained in the previous section were used as input of coRmiT.R ^18^, to detect correlated miRNA target gene pairs. In the case of this work, coRmiT.R applied several correlation strategies that calculate the Pearson correlation coefficient between miRNA and mRNA data. These correlations can be calculated using the following for miRNA or genes: (1) normalized counts for expressed genes/miRNA, (2) normalized counts for differentially expressed genes/miRNA and (3) using coexpression modules. In the latter case, we need a measure equivalent to the counts mentioned in the two previous cases. For this reason, we use the representation that WGCNA calculates about each identified coexpression module: the eigengene value or the hub gene profile. The eigengene is an average across all the members of the module (calculated for each analysed sample) and the hub gene profile is the miRNA/gen member with the highest correlation by this eigengene value. Each strategy is a permutation of one representation of miRNA expression and one representation of gene expression such as miRNA normalized counts versus gene normalized counts or eigengene miRNA versus gene hub for example. Then, a threshold of Pearson’s *R*<-0.8 was used to keep only negative associations resulting on as many sets of associated miRNA – target gene pairs as strategies. After that, odds ratio and Fisher’s exact test were calculated for the target genes of each miRNA-strategy combination to measure the matching with previously validated targets. The information about validated miRNA-targets was collected through the R package multimir^22^ that extracts data from the known experimentally databases miRecords^23^, miRTarBase^24^ and TarBase^25^. Moreover, the median odds ratio and coverage (ratio of miRNA with Fisher’s exact test FDR < 0.05) was calculated for each. As a high anticorrelation value do not ensures the proper miRNA – mRNA sequence complementarity, only previously validated miRNA-target pairs generated by the correlation strategy with higher median odds ratio were kept^22,23,25^. Enrichments for GO (http://geneontology.org/) terms and KEGG and Reactome pathways in miRNA-target validated targets was calculated using clusters_to_enrichment.R of ExpHunterSuite^21^. The original mRNA and miRNA sequencing data from mice at 50 weeks are available in the NCBI BioProject database under accession number PRJNA1232038.

## Results

### Identification of DEGs and DEMs in *Lmna*^*R249W*^ mutant hearts

**Fig. 1:**
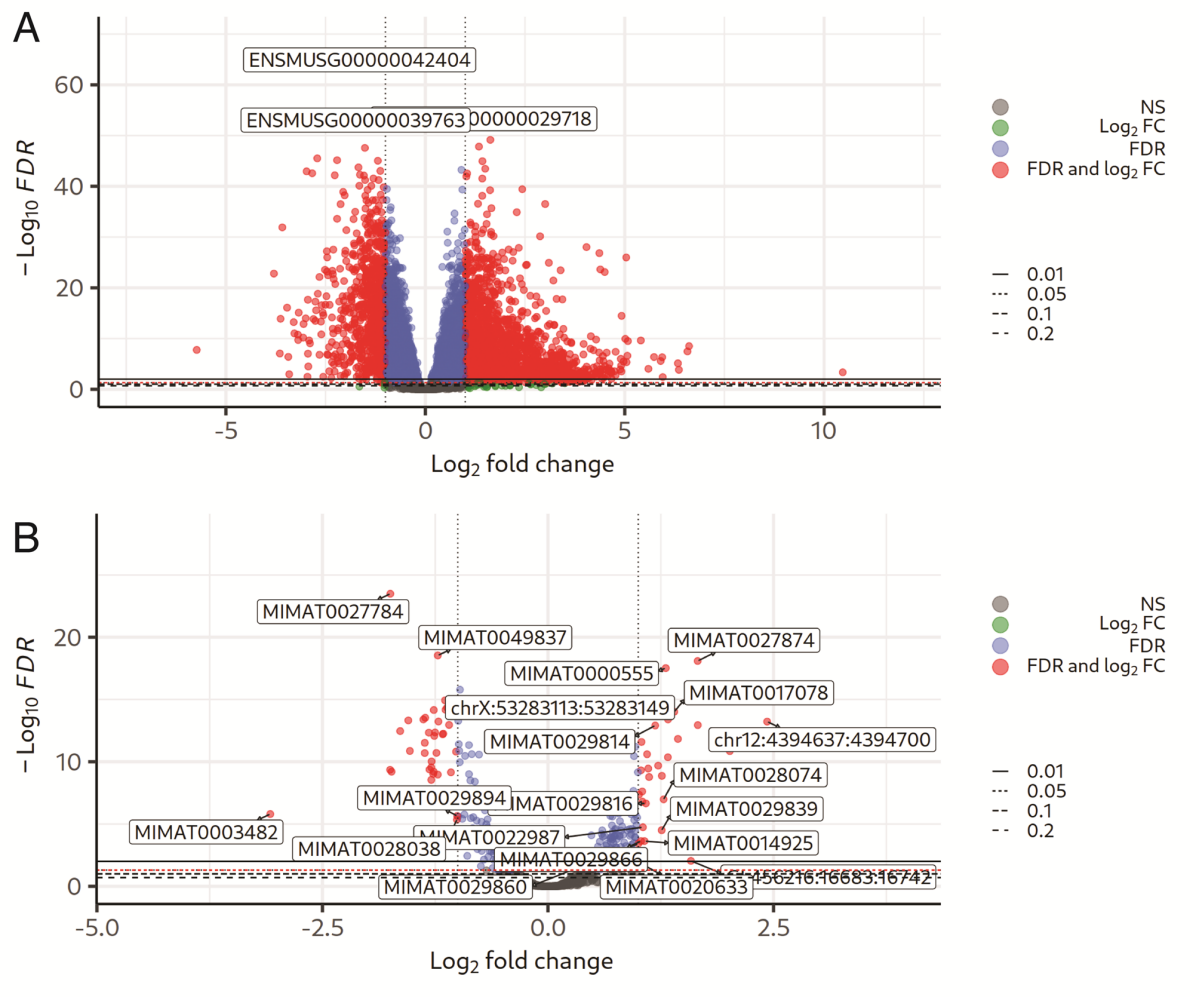
DEGs and DEMs in *LMNA*^*R249W*^ tissue samples. **(A)** The volcano plots showing DEGs in *LMNA*^*R249W*^ samples compared to wild type. **(B)** The volcano plots showing DEMs in *LMNA*^*R249W*^ samples compared to wild type. The X axis represents the log2 FC and the Y axis corresponds to -log FDR. The dots are genes/miRNA which have been classified in colors: red dots represent transcripts with |log2FC| > 1 and FDR < = 0.05, blue dots are those with |log2FC| <= 1 and FDR < = 0.05, green is those with |log2FC| > 1 and FDR > 0.05 and grey the remaining. Dashed lines show the different FDR thresholds. RNA was obtained from myocardial tissue of 50-week-old *Lmna*^*+/R249W*^ (*n* = 6) and age-matched wild-typed type mice (*n* = 6). Abbreviations: DEG differentially expressed genes; DEM: Differentially expressed micro-RNAs; FDR: false discovery rate; FC: fold change; log: logarithm; miRNA: micro RNA. https://drive.google.com/file/d/1-Ng6oPkTk6Zi48JRFrJP9Vaw3wX7RhcS/view?usp=drive_web.

To elucidate the mechanisms underlying DCM in *Lmna*^*R249W*^ heterozygous mutant mice, we analyzed both mRNA and miRNA expression profiles by RNA-seq in myocardial tissue of 50-week-old *Lmna*^*R249W*^ (*n* = 6) and age-matched wild-typed type mice (*n* = 6). Among 13,003 expressed genes, we identified 2,148 DEGs in *Lmna*^*R249W*^ hearts as compared to wild type, of which 1,485 were upregulated whereas 663 were downregulated (Fig. 1A and Supplementary File 1). We applied WGCNA to group the expressed genes in co-expression modules and modules 1, 2 and 3 showed the highest absolute correlation (Pearson |R| of 0.96, 0,96 and 0.87 respectively) with experiment design (Supplementary File 1). A total of 677 miRNAs were quantified of which 53 were differentially expressed (21 upregulated and 32 downregulated) in *Lmna*^*R249W*^ as compared to wild-type (Fig. 1B and Supplementary File 2).

### Functional and pathway enrichment analysis of DEGs

**Fig. 2:**
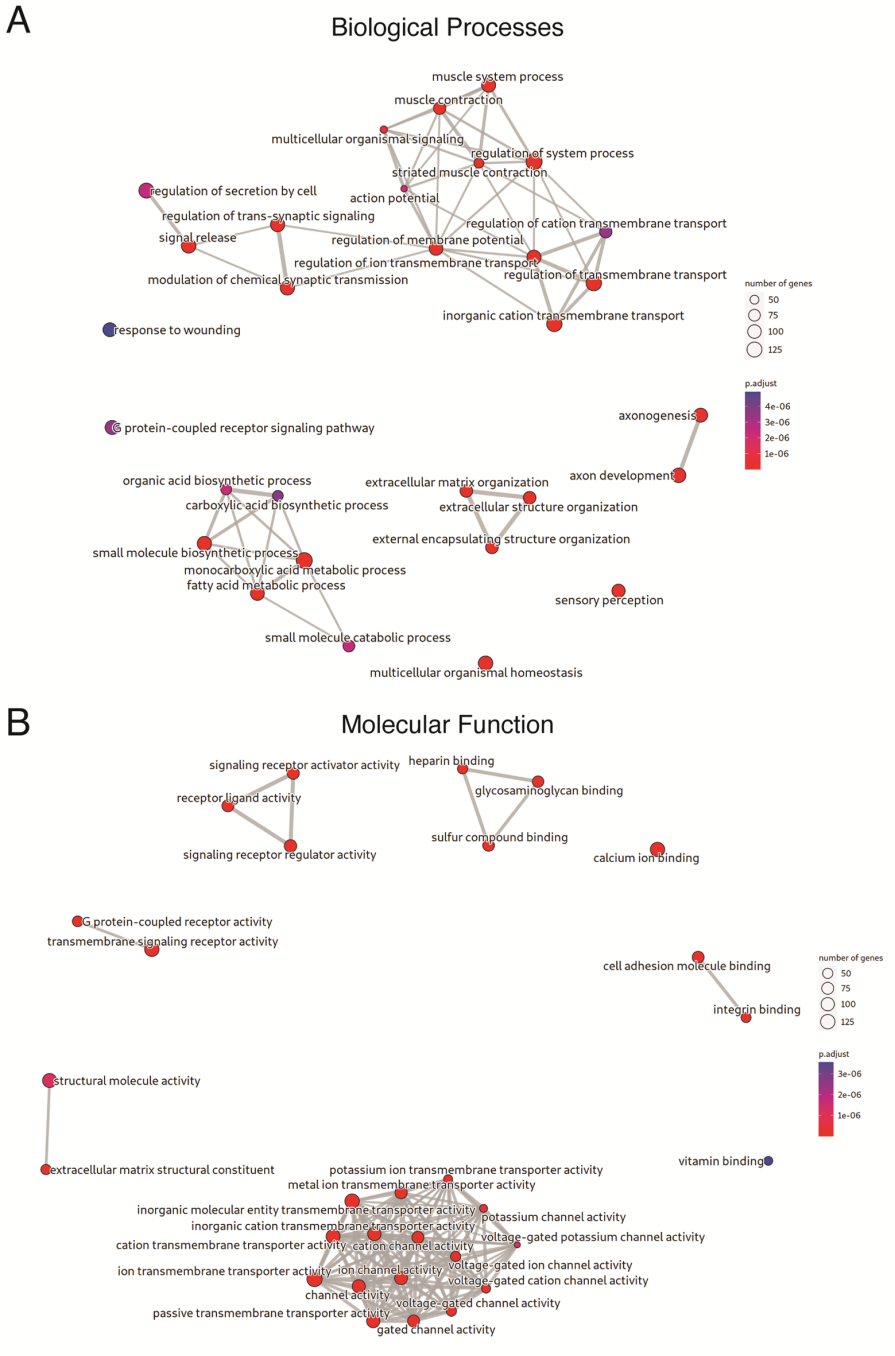
Enriched GO biological process and molecular function terms in differentially expressed genes. **(A)** Top 30 enriched GO Biological Process terms in DEGs. The color of the dots represents the FDR, and their size represents the number of DEGs annotated for each term. Two terms are linked when they shared annotated DEGs. The wider and shorter links correspond to more DEGs shared between terms. **(B)** Enriched GO Molecular Function terms in DEGs. Abbreviations: DEG differentially expressed genes; FDR: false discovery rate; GO: gene ontology. https://drive.google.com/file/d/1yt7qOhIeRaol9iQS_OloKMmhrpOvhbSu/view?usp=drive_web.

**Fig. 3:**
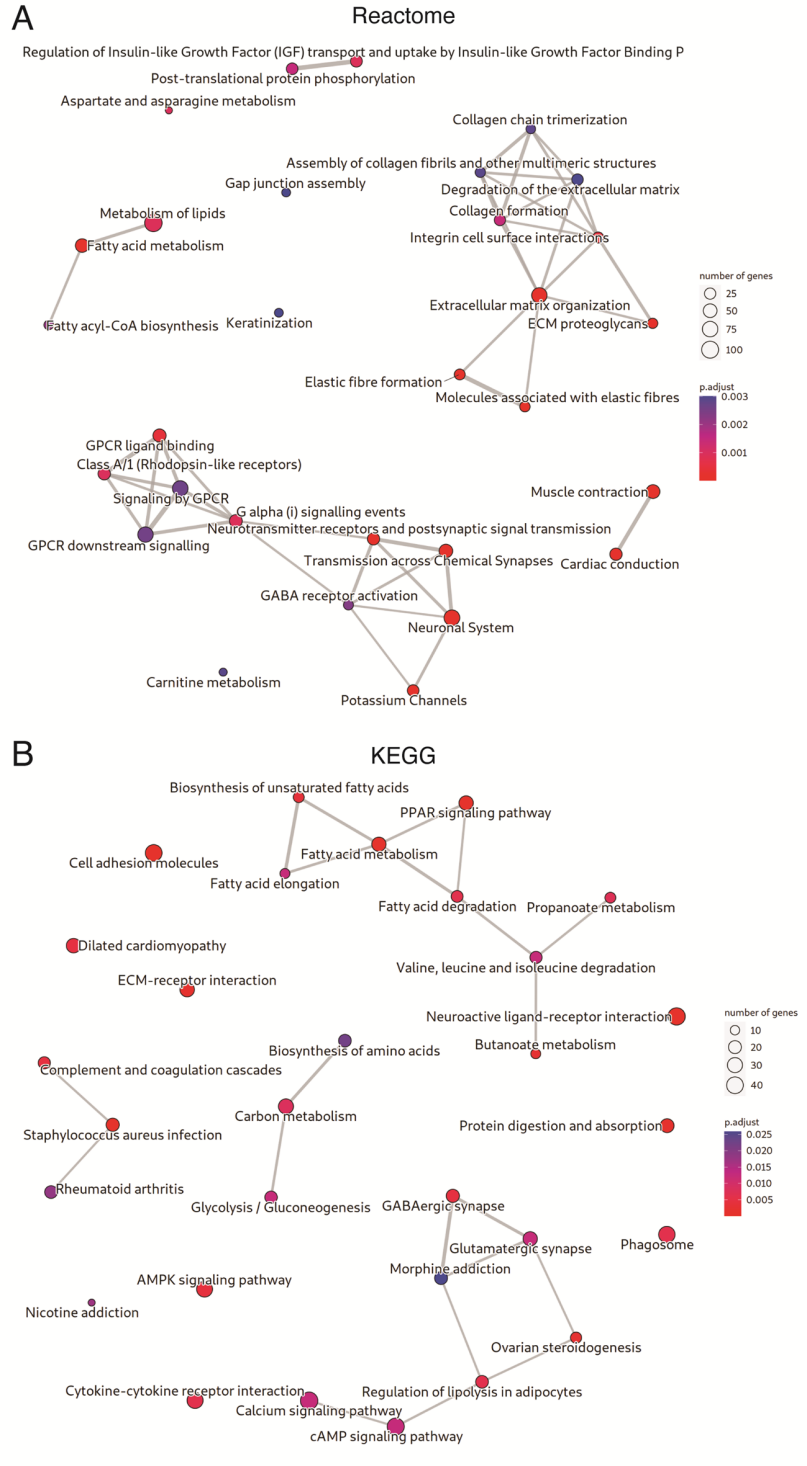
Enriched pathways in differentially expressed genes. **(A)** Reactome pathways. **(B)** KEGG pathways. This plot shows as dots the top 30 enriched pathways in DEGs. The color of the dots represents the FDR, and their size represents the number of DEGs annotated for each pathway. Two pathways are linked when they share annotated DEGs. The wider and shorter links correspond to more DEGs shared between pathways. Abbreviations: DEG: differentially expressed genes; FDR: false discovery rate; KEGG: Kyoto Encyclopedia of Genes and Genomes^96–98^. https://drive.google.com/file/d/1D2_7SHOTomN1Kk5ARRE5uhiTQB6KRjkN/view?usp=drive_web.

To better understand the mechanisms underlying *LMNA*-DCM, GO, KEGG and Reactome pathways analysis were conducted to predict the potential functions of DEGs. The most significantly enriched processes and signal pathways are shown in Figs. 2 and 3. Top enriched GO terms in biological processes (BP), molecular function (MF) and cellular components (CC) (Fig. 2A and B, and Supplementary File 3, respectively). The GO enriched terms were involved in fatty acid metabolic process, extracellular matrix, muscle contraction and synaptic transmission-related pathways among the main terms involved in BP (Fig. 2A). At the MF, DEGs were mainly enriched for voltage-gated ion channel-related pathways (Fig. 2B). Furthermore, extracellular matrix, sarcomere, synaptic transmission and ion channel-related terms of CC were significantly enriched (Supplementary File 3). Similarly, the enrichments for Reactome showed relations with lipid metabolism, extracellular matrix, muscle contraction and synaptic transmission-related pathways (Fig. 3A). Finally, KEGG pathway enrichments showed that DEGs were mainly involved in fatty acid metabolism-related pathway, cell adhesion molecules and dilated cardiomyopathy (Fig. 3B).

### MiRNA expression and target gene analyses

**Fig. 4:**
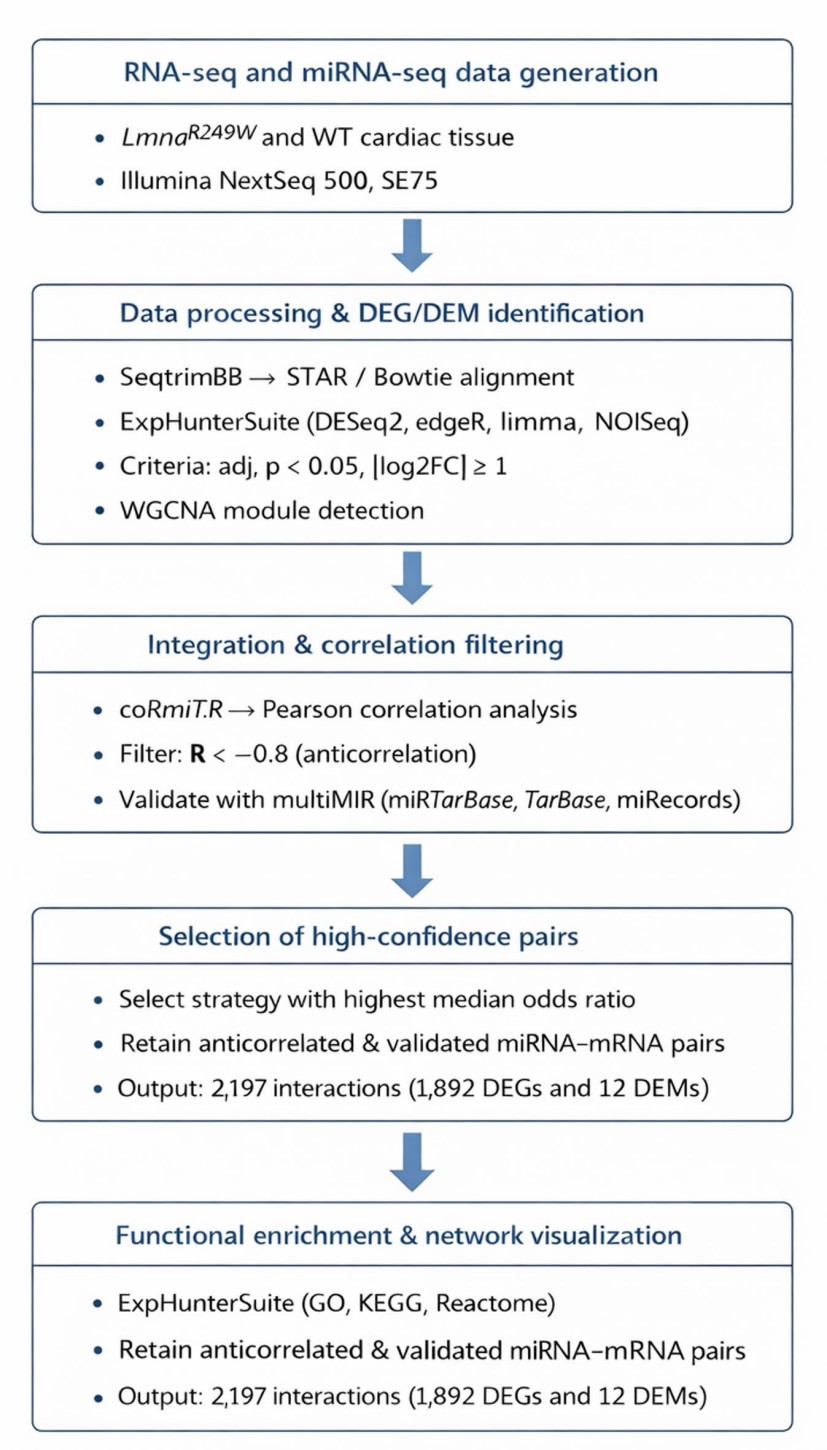
Integrative workflow for the identification of anticorrelated miRNA–mRNA pairs.

**Fig. 5:**
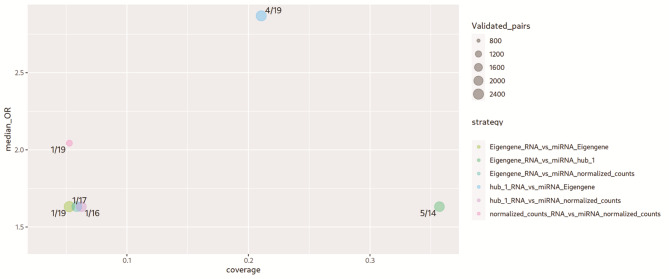
Comparison of miRNA–mRNA integration strategies based on median odds ratio (OR) and target coverage. This plot shows the ranking of the different correlation strategies by their odds ratio (Y axis) and their coverage (X axis). The higher odds ratio stands for more representation of the correlated pairs in database. The coverage is the proportion of miRNA with correlated targets within strategy that showed significant overlap with databases according to Fisher test FDR < 0.05. Dots represent the diverse correlation strategies where color distinguish the different analytical approaches. The strategies used are: (i) Correlation between differentially expressed mRNAs and miRNAs, using either opposite expression patterns (DEGs_RNA_vs_miRNA_DEMs_opp) or similar patterns (DEGs_RNA_vs_miRNA_DEMs_sim); (ii) Module-level integration, correlating mRNA and miRNA eigengenes (Eigengene_RNA_vs_miRNA_Eigengene) or restricting to hub genes within modules (Eigengene_RNA_vs_miRNA_hub_1); (iii) Gene-level correlations using normalized expression, either for all genes in the dataset (Eigengene_RNA_vs_miRNA_normalized_counts and normalized_counts_RNA_vs_miRNA_normalized_counts) or focusing on hub genes (hub_1_RNA_vs_miRNA_Eigengene and hub_1_RNA_vs_miRNA_normalized_counts). Labels indicate the number of validated interactions recovered relative to the total predicted for each strategy. Abbreviations: DEG differentially expressed genes; DEM: Differentially expressed micro-RNA; FDR: false discovery rate; FC: fold change; FDR: false discovery rate; miRNA: micro-RNA; OR: odd ratio. https://drive.google.com/file/d/1UIqgN-SdiIR5ZqBavNBsEDKExz6ekgcX/view?usp=drive_web.

To elucidate the regulatory role of DEMs in *Lmna*^*R249W*^ mutant hearts, the correlation between DEMs and DEGs and their associated co-expression modules was investigated by coRmiT. This tool associate miRNAs and genes following several correlation strategies. In this case, coRmiT was configured to associate only miRNAs and targets that correlate with a Pearson’s *R* < -0.8. Then, median odds ratio was used to determine which correlation strategy’s associations matches better with the validated miRNA target gene pairs from miRecords^23^, miRTarBase^24^ and TarBase^25^. The overall workflow for DEG/DEM integration and selection of anticorrelated miRNA–mRNA pairs is summarized in Fig. 4. The strategy that anticorrelates the eigengene value of miRNA modules with the hub gene profile of RNA modules showed the highest median odds ratio (2.87) and the second-best coverage, where 4 of 19 miRNAs had significant overlap FDR < 0.05 (Fig. 5). 108,676 miRNA-target anticorrelated pairs were found, of which 2,197 pairs matched with the previously validated. Those validated pairs correspond with only 12 miRNAs being four up-regulated DEMs (miR-183-5p, miR-690, miR-324-5p and miR-3473a) and eight down-regulated DEMs (miR-139-5p, miR-196b-5p, miR-3473b, miR-155-5p, miR-133a-5p, miR-1224-5p, miR-3095-5p, miR-149-5p). The functions of anticorrelated and validated targets were inspected using over representation analysis for GO terms. Only the targets of four upregulated (miR-183-5p, miR-3473a, miR-324-5p and miR-690) and eight downregulated miRNAs (miR-1224-p, miR-133a-5p, miR-149-5p, miR-155-5p, miR-196b-5p, miR-3095-5p, miR-3473b and miR-139-5p) showed significant enrichments for GO terms.

**Fig. 6:**
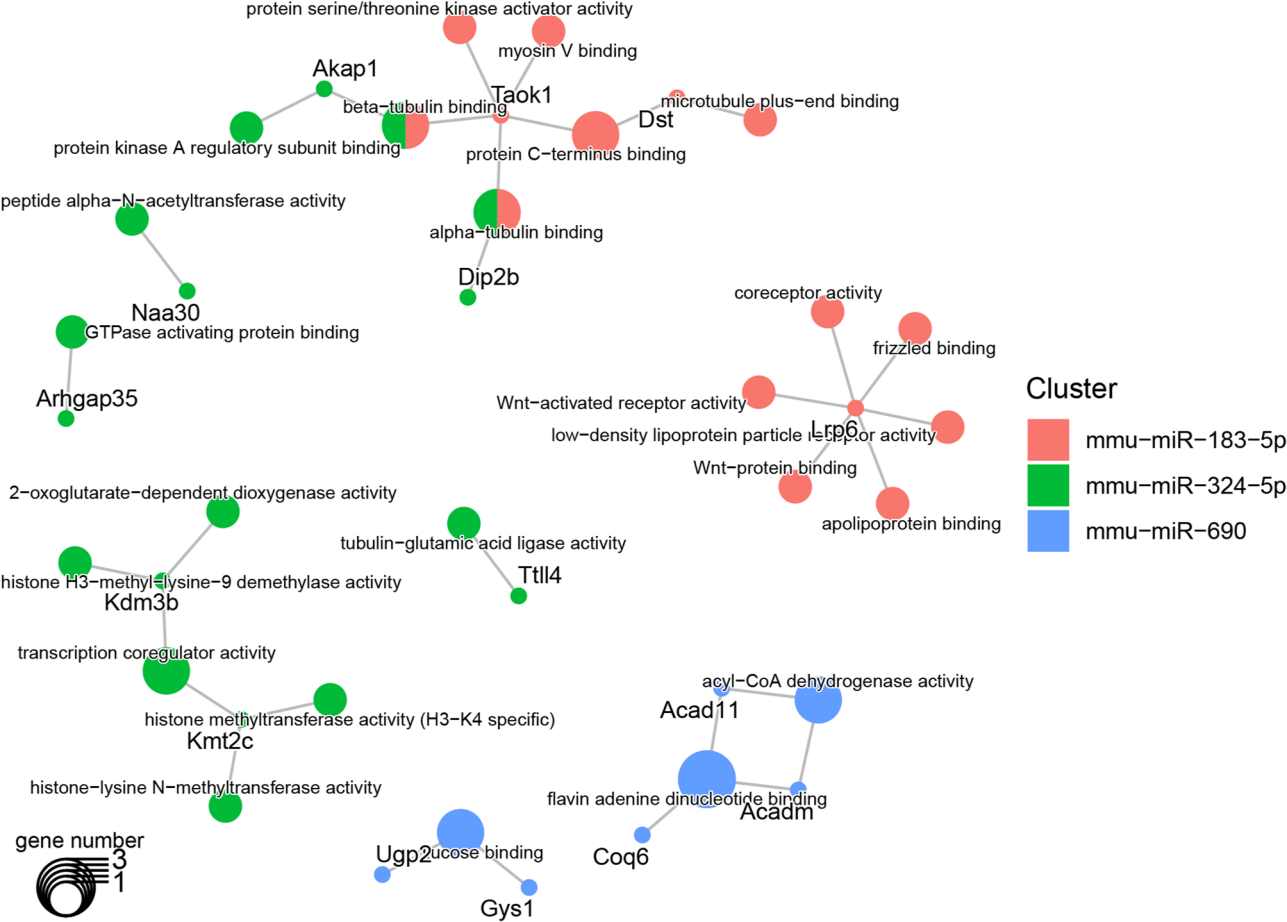
Enriched Gene Ontology Molecular Function terms in targets of the upregulated miRNA. This plot shows the significant enriched Gene Ontology Molecular Function terms (larger dots) in target genes (smaller dots). The color of the dots represents the miRNA that targets the gene and hence, the enriched term. The dots with two colors are targeted by two different miRNAs. The size of the term dot represents how many targets are annotated with it. Abbreviations: miRNA: micro-RNA. https://drive.google.com/file/d/1TnAszuM2gGV46APG6-o2kHAAI6SMIgcF/view?usp=drive_web.

**Fig. 7:**
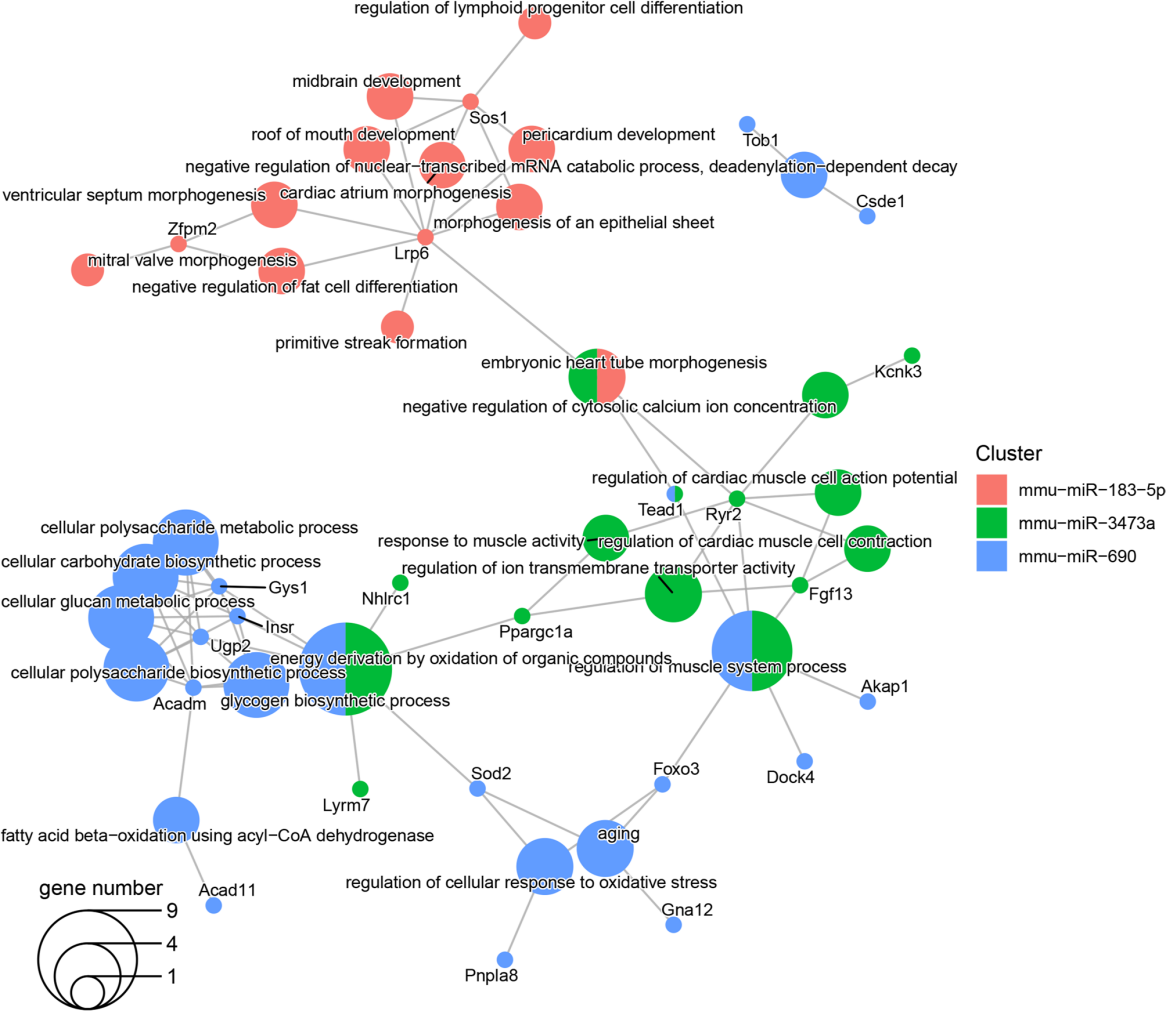
Enriched Gene Ontology Biological Process terms in targets of the upregulated miRNA. This plot shows the significantly enriched Gene Ontology Biological Process terms (larger dots) in target genes (smaller dots). The color of the dots represents the miRNA that targets the gene and hence, the enriched term. The dots with two colors are targeted by two different miRNAs. The size of the term dot represents how many targets are annotated with it. Abbreviations: miRNA: micro-RNA. https://drive.google.com/file/d/1tJhomdsp6oLoQ_paHhgYXd7WCXUSTN7y/view?usp=drive_web.

The targets of upregulated miRNAs using the most enriched terms of GO molecular functions and biological processes are shown in Figs. 6 and 7. The functional analysis demonstrated that the miR-183-5p targets *Lrp6*,* Zfmp2* and *Sos1* are involved in crucial processes of the developing heart including mitral valve, ventricular septum, cardiac atrium and embryonic heart tube morphogenesis, and pericardium development. Whereas the miR-183-5p target Lrp6 was related to the Wnt-signaling pathway and apolipoprotein binding, *Taok1* and *Dst* were associated with myosin V-, α-tubulin- and β-tubulin-binding. Similarly to miR-183-5p, the miR-324-5p targets *Akap1* and *Dip2b* affecting α-tubulin and β-tubulin binding. Other miR-324-5p targets such as *Arhgap35*, *Naa30* and *Kmt2c/Kdm3b* were involved in GTPase binding, peptide α-N-acetyltransferase activity and histone methylation/demethylation function (Figs. 6 and 7).

The miR-3473a targets were related to embryonic heart tube morphogenesis, regulation of cardiomyocyte action potential and cardiac muscle cell contraction through the *Ryr2* gene; this former shares *with* the *Kcnk3* gene the negative modulation of cytosolic calcium ion concentration. *Ryr2* and *Tead1* genes are linked to the embryonic heart tube morphogenesis and, control muscle system process. The regulation of ion transmembrane transporter activity and response to muscle activity is performed targeting *Ryr2* and *Ppargc1a* and, the energy derivation by oxidation of organic compounds through *Ppargc1a*,* Lyrm7* and *Nhlrc1*. The targets for miR-690 were enriched for terms affected functions as the control of muscle system process, the positive regulation of reactive oxygen species biosynthetic process, the aging, the regulation of cellular response to oxidative stress, the energy derivation by oxidation of organic compounds and, the fatty acid β-oxidation using acyl-CoA dehydrogenase; some of these functions were shared with miR-3473a, but just the *Tead1* gene (Fig. 7). The miR-690 targets were also enriched for terms related to glucose binding, and acyl-CoA dehydrogenase activity (Fig. 6).

**Fig. 8:**
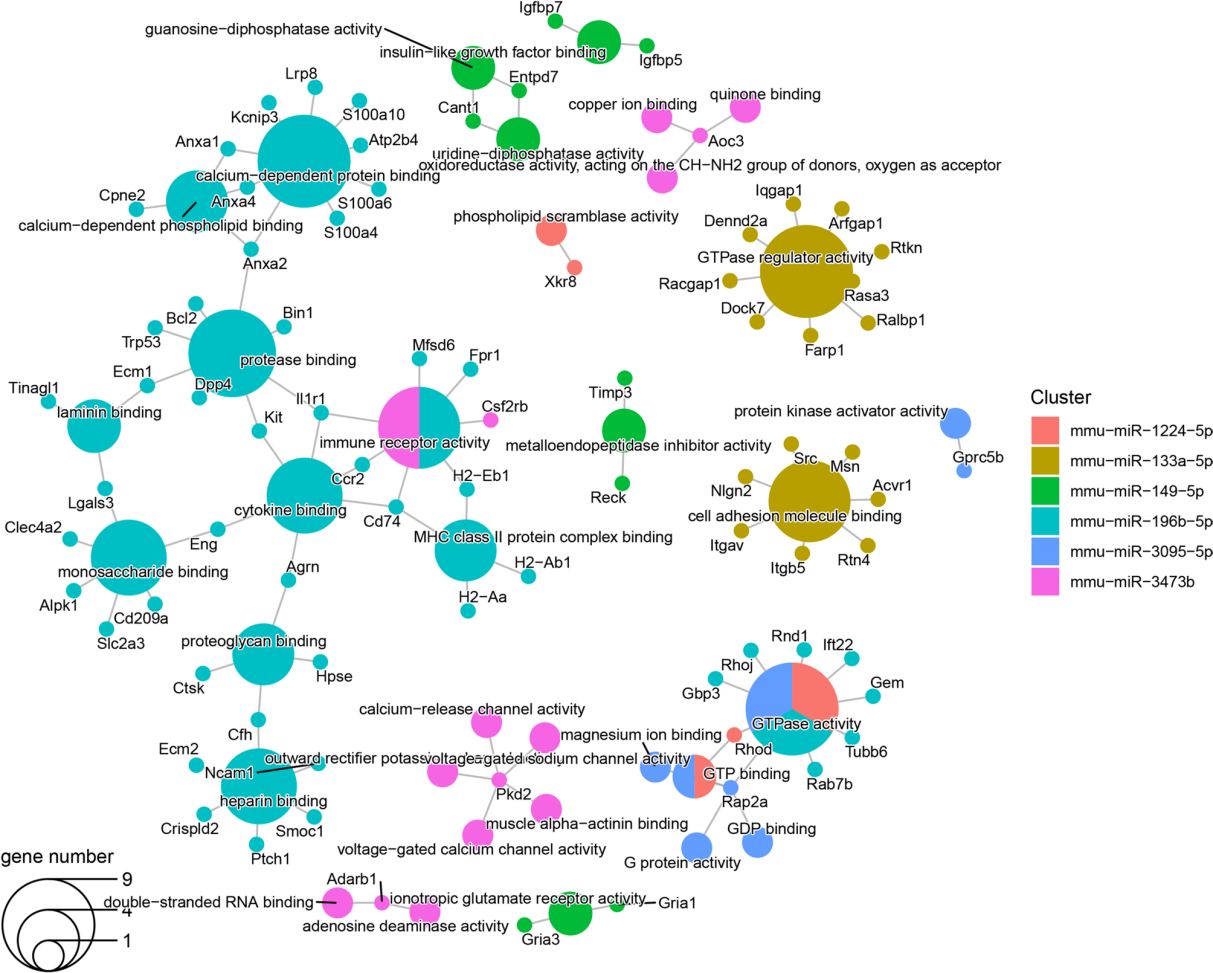
Enriched Gene Ontology molecular function terms in targets of the downregulated miRNAs. This plot shows the significantly enriched Gene Ontology Molecular Function terms (larger dots) in target genes of each miRNA (smaller dots). The color of the dots represents the miRNA that targets the gene and hence, the enriched term. The dots with two colors are targeted by two different miRNAs. The size of the term dot represents how many miRNAs targeted genes annotated with each term. Abbreviations: miRNA: micro-RNA. https://drive.google.com/file/d/1jMLjuHDEjawPyAo7sIG8VD81wHWUVOQd/view?usp=drive_web.

**Fig. 9:**
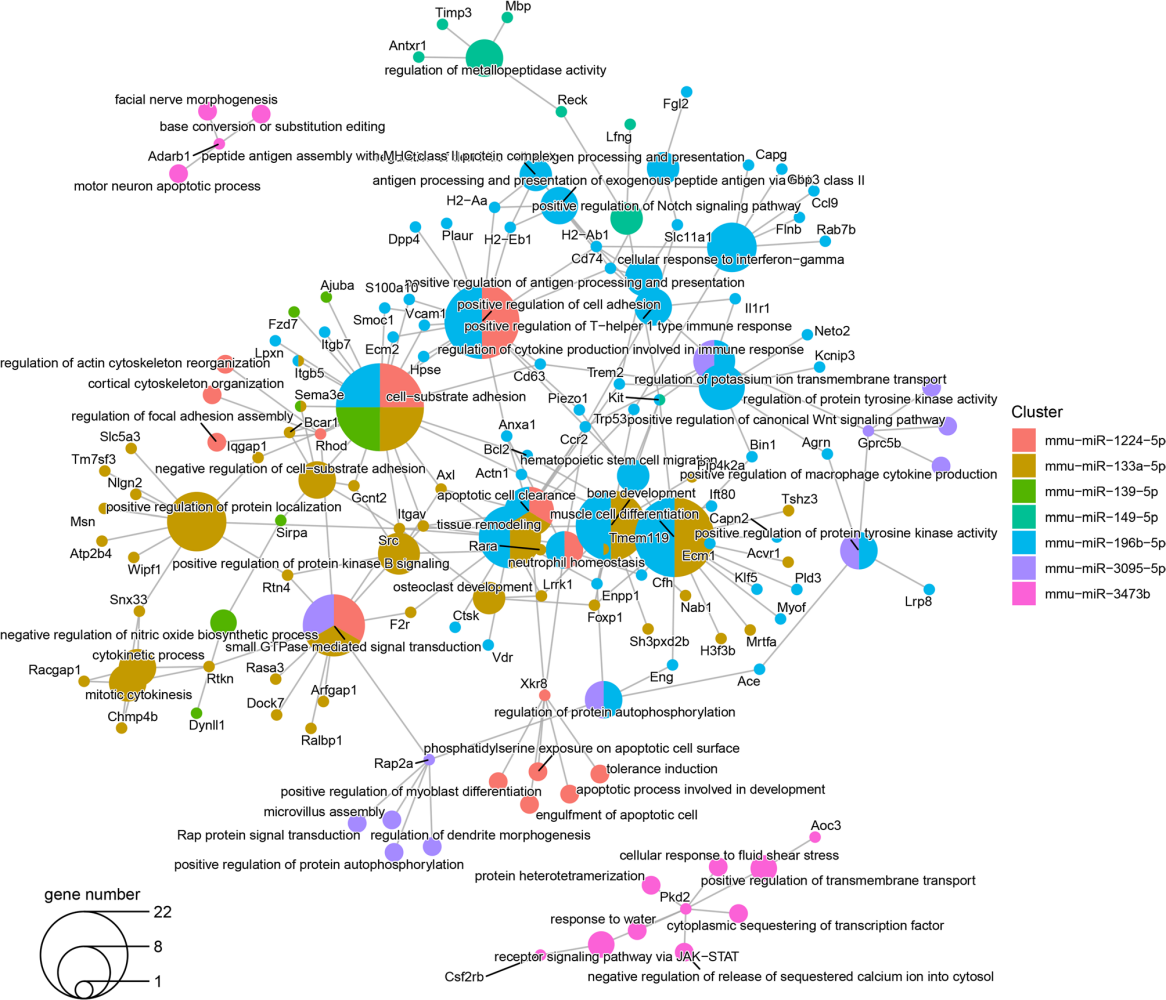
Enriched Gene Ontology Biological Process terms in targets of the downregulated miRNA. This plot shows the significant enriched Gene Ontology Biological Process terms (larger dots) in target genes of each miRNA (smaller dots). The color of the dots represents the miRNA that targets the gene and hence, the enriched term. The dots with two colors are targeted by two different miRNAs. The size of the term dot represents how many miRNAs targeted genes annotated with each term. Abbreviations: miRNA: micro-RNA. https://drive.google.com/file/d/1xPuPJgeH_a4wsRUF04I-FNWIDa6A7IQs/view?usp=drive_web.

Similarly, the downregulated miRNAs´ targets using the most enriched terms of GO MF and BP are presented in Figs. 8 and 9. Among them, the targets of five downregulated miRNAs, miR-1224-5p, miR-133a-5p, miR-139-5p, miR-155-5p and miR-196b-5p involved the cell-substrate adhesion term, whereas cell-matrix adhesion and apoptotic cell clearance were enriched by targets of miR-1224-5p, miR-133a-5p, miR-155-5p and miR-196b-5p. miR-1224-5p, miR-155-5p and miR-196b-5p also affected the positive regulation of cell adhesion and actin filament organization cytoskeleton. In addition, miR-1224-5p and miR-155-5p targets were enriched for terms related to the regulation of actin cytoskeleton reorganization, the regulation of focal adhesion, cortical cytoskeleton organization, regulation of cell-substrate adhesion and apoptotic process involved in development. Besides, miR-155-5p and miR-196b-5p targets also were enriched for terms associated with inflammatory response, wound healing, extracellular matrix organization and muscle cell differentiation. The miR-155-5p and miR-196b-5p target genes were involved in laminin, protease, actinin filament, MHC class II and calcium binding pathways related terms. miR-155-5p together miR-149-5p targets were implicated in the Notch-signaling pathway and metallopeptidase activity regulation, and targets shared by miR-155-5p and miR-1224-5p were involved in the terms GTP binding and lipid transporter function. Finally, miR-3473b targets were associated with calcium and sodium channel activity-related pathways and muscle alpha-actinin binding.

### Validation of DEMs by RT-qPCR

**Fig. 10:**
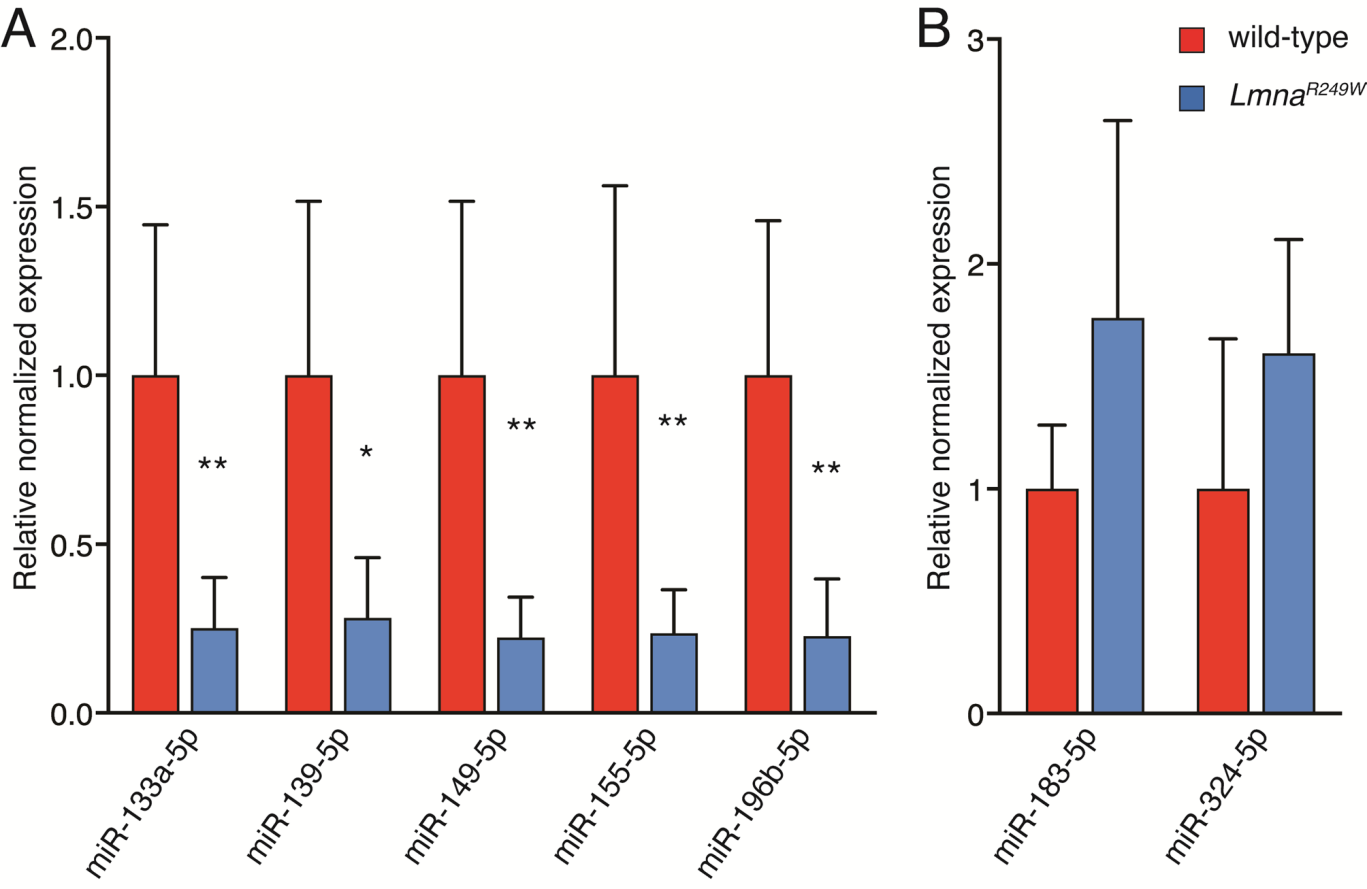
A qRT-PCR validation. Expression levels of miRNAs in *LMNA*^*R249W*^ (*n* = 6) vs. wild-type (*n* = 6) analyzed by qRT-PCR. (A) Downregulated miRNAs. (B) Upregulated miRNAs. Statistical significance was determined using a two-tailed t test. * *p* < 0.05; ** *p* < 0.01. Abbreviations: miRNA: micro-RNA. https://drive.google.com/file/d/13IkSMdaSrOY7ykTe5zW4GkmAxAYw1MiK/view?usp=drive_web.

We performed experimental validation using qRT-PCR for certain DEMs, such as miR-133a-5p, miR-139-5p, miR-149-5p, miR-155-5p, miR-183-5p, miR-196b-5p and miR-324-5p. These miRNAs were selected based on their correspondence to validated targets identified through miRNA-target correlated pairs analysis, which encompassed 12 DEMs. Among these DEMs, we prioritized those for which commercial primers were available. qRT-PCR were performed in six *Lmna*^*R249W*^ and six wild-type myocardial samples. U6 and 5s were used as internal control for miRNA qRT-PCRs. For qRT-PCR confirmation, all seven DEMs showed a strong correlation between RNA-seq and qRT-PCR (Fig. 10 and Supplementary File 2).

## Discussion

We have performed for the first time a comprehensive characterization of the mRNA and miRNA transcriptome of myocardial tissue from *Lmna*^*R249W*^ mutant mice which develop DCM as an isolated phenotype. Although transcriptome works in the heart of *Lmna* mutant mice have been reported in the past^26–31^, our study is the first combining small RNA and mRNA sequencing to investigate miRNA-target interactions in an in vivo setting of *LMNA*-DCM to unveil the molecular mechanisms behind the pathogenesis and progression of this condition. We report that *Lmna*^*R249W*^ hearts display distinct mRNA and miRNA expression profiles with 2,148 DEGs and 53 DEMs. We found that DEGs were mainly involved in cardiac-related pathways such as extracellular matrix, muscle contraction, voltage-gated ion channel- and synaptic transmission-related pathways, cell adhesion molecules and fatty acid metabolic process. At this point, we should mention a limitation of our study regarding the use of a mouse model and a single time point. We also want to point out that further experimental studies should be carried out to replicate our results in human tissues to translate the data obtained into clinical practice.

Myocardial fibrosis, a principal adaptative response in DCM, acts through extracellular matrix changes^32^. The DEGs in *Lmna*^*R249W*^ hearts were enriched for extracellular matrix in line with recent studies that demonstrated the upregulation of extracellular matrix genes in the heart of distinct *Lmna* mutant mouse lines^33,34^. Extracellular proteins impairment may alter cell-to-cell interactions which impact the mechanical cues modifying signaling pathways. This mechano-transduction stimulus may lead to a maladaptive remodeling response in *LMNA*-DCM^35^. DCM drives to an abnormal hemodynamic load and mechanical stress to respond to the gene expression, protein synthesis and cardiomyocyte degradation cascade^36^.

Besides ventricular dilatation and remodeling, *LMNA*-DCM is also characterized by conduction defects and life-threatening arrhythmogenic events, sometimes before any detectable left ventricular impairment^37,38^. Voltage-gated ion channels are responsible for action potential generation and its propagation across the myocardium, mainly sodium^39^. Focusing on *LMNA*-DCM, several studies have linked *LMNA* deleterious variants to *SCN5A* regulation and Nav1.5 function^40–44^. In recent research, a deleterious variant in the *LMNA* gene was associated with hyper-polymerization and hyper-acetylation of the tubulin network with concomitant downregulation of Nav1.5 cell expression and activity, leading to disruption of electric transmission^40^. This incorrect communication between cardiomyocytes induces Cx43 remodeling in ventricles^45^, and high risk of arrhythmias, hallmark in *LMNA*-DCM. In the same way, our results showed an enrichment of voltage-gated ion channel-related pathways in *Lmna*^*R249W*^ hearts, accordingly to dysregulation of genes that encode sodium voltage-gated channel subunits in the heart from a different *Lmna* mutant mouse line^29^. On the other hand, although the role of cardiac neurotransmitter system in DCM has not been fully investigated, alterations of presynaptic sympathetic innervation have been associated with idiopathic DCM^46^. Hence, our results show, for the first time, that cardiac synaptic transmission might be implicated in *LMNA*-DCM.

Desmosomes are critical adhesion structures in cardiomyocytes that mediate strong cell-cell contact^47^. While pathogenic variants encoding desmosomal proteins are considered the predominant cause of arrhythmogenic cardiomyopathy^48^, pathogenic variants in the *LMNA* gene have also been associated to be possible causes of this disease, especially with severe bradyarrhythmia^49–51^. The pathophysiological mechanisms underlying the arrhythmic phenotype in *LMNA*-DCM are still not well elucidated but the structural nuclei abnormalities, chromatin modifications and the associated transcriptional changes may influence the molecular basis of the *LMNA*-related cardiac phenotypes, including arrhythmogenic events^52^. It has been suggested that the loss of A-type-lamins could have an impact on cytoskeleton organization and cell adhesion^53^. Accordingly, our analysis showed an involvement of DEG genes in cell-cell adhesion. The deregulation of key genes involved in fatty acid metabolism are related to DCM^54,55^, but its involvement in *LMNA*-DCM remains unknown. Hence, we confirm that fatty acid metabolism impairment might be implicated in the pathogenesis of *LMNA*-DCM.

Integrative analysis identified 2,197 validated interactions between 1,892 DEGs and 12 DEMs. Enrichment analysis of target genes for each upregulated miRNA showed that genes regulated by miR-183-5p highlighted heart development processes, and myosin V-, α-tubulin- and β-tubulin-binding, suggesting a previously undescribed role of miR-183-5p in the developing heart. Consistently with the enrichment for myosin V-, α-tubulin- and β-tubulin-binding, Tariq et al. suggested that appropriate nuclear lamina organization and microtubule network are required for maintaining an adequate nuclear morphology and function^56^. In this regard, Borin et al. found that pathogenic variants in *Lmna* compromise the microtubule network in neonatal rat ventricular myocytes^57^. According to our analysis, miR-183-5p targets *Lrp6*, a gene encoding the coreceptor to Frizzled in the Wnt pathway^58^, which is consistent with previous results, demonstrating that dysregulation of Wnt/β-catenin pathway and its downstream target gap junction protein connexin-43 contributes to the pathophysiology of DCM in *Lmna* mutant hearts^59–61^. Therefore, miR-183-5p might be involved in *LMNA*-related malignant arrhythmias leading to electrical conduction disturbances consequence of an incorrect communication between cardiomyocytes due to alterations in microtubule cytoskeleton, acetylation of α-tubulin and subsequent Cx43 remodeling^45^. In the same way occurs in the *Lmna*^*tm1Stw*^ mutant mouse line, in which LRP6 deficiency led to DCM due to alterations in the autophagic degradation and fatty acid utilization pathways^26,29^.

The target genes of miR-324 were involved in histone methylation/demethylation function which is consistent with the role of Lamin A protein in the epigenomic regulation of chromatin^62^. In addition, its target gene *Atg4* is an autophagic-related gene involved in removing damaged products of the cell, and recycling proteins, glycogen, and fatty acids, thus providing energy for myofibers during stress and/or energy deprivation^63^. Hence, miR-3473a is a principal regulator of heart development and, is involved in heart failure^64^. Our analysis confirms these results. miR-3473a target genes highlighted cardiac muscle cell action potential and contraction, negative regulation of cytosolic calcium ion concentration, regulation of ion transmembrane transporter activity and, response to muscle activity via Ryanodine receptor 2 (*Ryr2*). Abnormal ryanodine function is present in inherited cardiac arrhythmias known as cardiac ryanodinopathies, mainly catecholaminergic polymorphic ventricular tachycardia^65^. Dridi et al. reported biochemical modification of RyR2 protein in the heart tissue in both human patients with *LMNA*-DCM and *Lmna* mutant mice^66^. Similarly to miR-183-5p, miR-3473a could be involved in the progression of arrhythmic events in *LMNA*-DCM. Our analysis showed the transcription factor-encoding gene *Tead1*, a critical component of the Hippo signaling pathway together with the coactivator YAP/TAZ^67^, as a potential target of both miR-3473a and miR-690. Agreeing with this, *Tead1* knockout mice showed embryonic lethality and DCM^68^, whereas specific ablation of *Tead1* in adult cardiomyocytes after tamoxifen induction led to lethal acute-onset DCM^69^. Hippo signaling activation/YAP-TEAD1 inactivation leads to mitochondrial damage promoting DCM^70^. More recently, single-cell RNA-seq experiments confirmed dysregulated expression of TEAD1 target genes in cardiac tissue from patients with *LMNA*-DCM but not in other DCM patients^71^.

Oxidative stress has been linked strongly to cell death and cardiac remodeling processes, both leading to heart failure, being antioxidant treatment a therapeutic approach for cardiomyopathies^72^. miR-690 target genes were also involved in positive regulation of reactive oxygen species biosynthetic process and regulation of cellular response to oxidative stress targeting *Sod2*. Superoxide dismutase (SODs) is antioxidative enzymes that catalyze the degradation of reactive oxygen species. Therefore, they regulate mitochondrial superoxide generation and improve the phenotypes of the DCM and muscle fatigue in mice^73^. In line with this, alterations of SOD2 protein have been linked to DCM progression and ventricular tachycardia both in mice and humans^74,75^.

Regarding the downregulated miRNAs, our analysis showed that many of these miRNAs were enriched in linked BP and MF terms. Concretely, miR-1224-5p, miR-133a-5p, miR-139-5p, miR-155-5p and miR-196b-5p shared the cell-substrate adhesion term, whereas miR-1224-5p, miR-133a-5p, miR-155-5p and miR-196b-5p shared cell-matrix adhesion and apoptotic cell clearance. Many other terms were shared by at least two miRNAs. Prior studies have shown that a mRNA can be targeted by multiple miRNAs simultaneously and regulate the same transcript targets^76^. The synergistic effects of miRNAs are important for distinct biological processes^77–79^, including key pathways related to cardiac diseases^80,81^. miR-1224-5p was mainly involved in the regulation of BP associated with cell adhesion, cytoskeleton organization and apoptosis. Its role in the heart has yet to be explored but, miR-1224-5p has been described to suppress apoptosis and epithelial-to-mesenchymal transition via TGF-ß1/Smad3 signaling pathway; this former is a process characterized by loss of cell-cell adhesions and polarity and the reorganization of the cytoskeleton^82,83^, which might indicate a regulatory role in cell invasion and apoptosis in the heart. In addition, an impairment in the TGF-β pathway has been recently described in DCM patients^36^. Regarding miR-133a-5p, similar findings were reported in other forms of DCM as the miR-133 family was downregulated in cardiac tissue from patients with this condition^84^. Furthermore, studies have demonstrated a role for miR-133a-5p in the pathology of ischemic myocardial diseases inhibiting apoptosis, inflammation, and adverse cardiac remodeling^85^, which agrees with the enrichment of the apoptotic cell clearance^86^. The role of miR-139-5p in the heart remains poorly understood; according to our results, its expression is downregulated in the myocardial tissue from patients with both ischemic and hypertrophic cardiomyopathy^87^. miR-139-5p that acts as an anti-hypertrophic miRNA attenuating cardiomyocyte enlargement is involved in *LMNA*-DCM biological process^88^. miR-149-5p in the heart has been linked in vitro studies to cardiomyocyte apoptosis^31^. Our results identified miR-149-5p as a potential regulator of the Notch pathway, intercellular signaling that regulates cell fate specification and organogenesis^89^. Both aberrations in Notch signaling and pathogenic variants in *LMNA* are related to left ventricle non-compaction cardiomyopathy^41,90^. Recently, emerging data have indicated a role of Notch pathway with crosstalk with Hippo signaling in the progression of inherited DCM^91^. In the heart, miR-155-5p was upregulated in inflammatory DCM^92,93^, being critical for immune response in the myocardium^94^. *LMNA*-DCM is characterized by a dysregulated inflammatory response along the contractile and electrical impairment^95^, nevertheless, our results showed downregulation of miR-155-5p in *Lmna*^*R249W*^ hearts. Hence, further studies are needed to understand the role of miR-155-5p in this entity. Finally, to date, nothing is known about the role of miR-196b-5p. Our results show for the first time a dysregulation of these miRNAs in *LMNA*-DCM suggesting a role in heart disease.

From a translational perspective, among the extensive network of miRNA–mRNA pairs identified, miR-183-5p/LRP6 and miR-3473a/RYR2 emerge as particularly relevant candidates. Both pairs involve components previously implicated in pathways central to LMNA-DCM pathophysiology (Wnt/β-catenin signaling and calcium-handling regulation) which are recurrently altered in dilated and arrhythmogenic cardiomyopathies^40,65^. On other hand, miR-183-5p and miR-3473a have also been implicated in cardiac development and oxidative stress responses in independent studies^64^, further supporting their functional relevance. Their consistent appearance across distinct cardiac remodeling processes highlights them as potential upstream regulatory nodes warranting further validation in human tissue.

This study is limited by its in-silico nature, as the identification of miRNA–mRNA regulatory interactions was based on computational integration of expression and correlation data. Although experimental validation would strengthen these findings, the large number of predicted and validated interactions (> 2,000) makes such an approach unfeasible within the scope of the present work. Instead, our results provide an integrative framework to prioritize the most biologically relevant candidates for future targeted validation studies (Table 1).

In summary, we must note that the gene expression related to pathophysiological changes at different stages of *LMNA* cardiomyopathy will be performed in the next step. Further studies in larger murine cohorts should be achieved to confirm our results and therefore, translate our first in vivo approach to clinical studies of *LMNA*-DCM patients. In summary, our study explores, for the first time, the molecular mechanisms behind *LMNA*-DCM through the integration of mRNA and miRNA sequencing data. This integrative approach allowed us to identify novel miRNA-mRNA interaction networks and signaling pathways that unravel cellular biological processes of *LMNA*-DCM. We suggest our results will open new avenues for the biomarkers research in *LMNA*-MCD and might be used as novel therapeutic targets for treating *LMNA*-related DCM.

**Table 1:** miRNA and summary of their targets.

miRNA	miRNA log2FC	Validated targets	RNA-seq modules
mmu-miR-690	1.66	46	1,10
mmu-miR-183-5p	1.33	9	1,10
mmu-miR-324-5p	1.30	9	1,10
mmu-miR-3473a	1.12	21	1,10
mmu-miR-182-5p	2.01	0	5
mmu-miR-758-5p	1.44	0	1,10
mmu-miR-375-5p	1.40	0	1
mmu-miR-5619-5p	1.10	0	1,10
mmu-miR-139-5p	-1.08	22	2,3,39
mmu-miR-196b-5p	-1.10	199	2,3,39
mmu-miR-3473b	-1.14	6	2,3,39
mmu-miR-155-5p	-1.26	1718	2,3,39
mmu-miR-133a-5p	-1.27	109	2,3,39
mmu-miR-1224-5p	-1.32	2	2,3,39
mmu-miR-3095-5p	-1.38	2	2,3,39
mmu-miR-149-5p	-1.53	61	2,3,39
mmu-miR-1249-5p	-1.16	0	2,3,39
mmu-miR-133b-5p	-1.29	0	2,3,39
mmu-miR-5132-5p	-1.36	0	2,3

This table summarizes the coRmiT output showing the DEMs with correlated targets, their log2FC. The table also shows how many correlated targets of each miRNA have been previously validated (according to databases) and the RNA-seq co-expression modules that include those targets. Abbreviations: DEM: differentially expressed miRNA; mmu: mus muscullus; miRNA: micro-RNA.

## Supplementary Information

Below is the link to the electronic supplementary material.

Supplementary Material 1

## Data Availability

Data transparency is guaranteed. The datasets generated during and/or analyzed during the current study are available in the supplemental material. The original mRNA and miRNA sequencing data from mice at 50 weeks are available in the NCBI BioProject database under accession number PRJNA1232038.
