# Correction: An integrative approach to identify novel miRNA-mRNA interaction networks in *LMNA-*cardiomyopathy

**DOI:** 10.1038/s41598-026-45043-w

**Published:** 2026-03-31

**Authors:** José Córdoba-Caballero, Fernando Bonet, Oscar Campuzano, Georgia Sarquella-Brugada, Ignacio Perez de Castro, Borja Vilaplana-Martí, Pedro Seoane-Zonjic, Alipio Mangas, Juan A. G. Ranea, Rocio Toro

**Affiliations:** 1 Biomedical Research and Innovation Institute of Cadiz (INiBICA), Research Unit, Puerta del Mar University Hospital, Cádiz, Spain; 2 Department of Molecular Biology and Biochemistry, University of Málaga, Malaga, Spain; 3 H12O-CNIO Haematological Malignancies Clinical Research Unit, Spanish National Cancer Research Centre, Madrid, Spain; 4 Medicine Department, Medical School of Cádiz, Cádiz University, 11003 Cádiz, Spain; 5 Medical Science Department, School of Medicine, University of Girona, Girona, Spain; 6 Institut d’Investigació Biomèdica de Girona (IDIBGI-CERCA), Salt, Spain; 7 Centro Investigación Biomédica en Red, Enfermedades Cardiovasculares (CIBERCV), Madrid, Spain; 8 Pediatric Arrhythmias, Inherited Cardiac Diseases and Sudden Death Unit, Cardiology Department, Sant Joan de Déu Hospital, Barcelona, Spain; 9 Arrítmies Pediàtriques, Cardiologia Genètica i Mort Sobtada, Malalties Cardiovasculars en el Desenvolupament, Institut de Recerca Sant Joan de Déu, Barcelona, Spain; 10 Instituto de Investigación de Enfermedades Raras , Instituto de Salud Carlos III, Madrid, Spain; 11 Servicio Cirugía Experimental, Instituto Aragonés Ciencias de la Salud (IACS), Zaragoza, Spain; 12 Department of Molecular Biology and Biochemistry, University of Málaga, Malaga, Spain; 13 Internal Medicine Department, Puerta del Mar University Hospital, Cádiz, Spain; 14 Institute of Biomedical Research in Málaga (IBIMA Plataforma BIONAND), Malaga, Spain

Correction to: *Scientific Reports* 10.1038/s41598-026-36439-9, published online 24 January 2026

The original version of this Article contained an error in the name of author Fernando Bonet which was incorrectly given as Fernando Bonet Martínez.

As a result, the Equal contribution Statement,

“José Córdoba-Caballero, Fernando Bonet Martínez, contributed equally to this work.”

now reads:

“José Córdoba-Caballero, Fernando Bonet, contributed equally to this work.”

The original Article has been corrected.

