# Supplementary material for: An integrative approach to identify novel miRNA-mRNA interaction networks in LMNA-cardiomyopathy
Source: Sci Rep. 2026 Jan 24;16:6110. doi: 10.1038/s41598-026-36439-9 (PMC12901163; doi:10.1038/s41598-026-36439-9)
Supplement: Supplementary file 1 — Supplementary Material 1 [file 41598_2026_36439_MOESM1_ESM.docx]

**Supplemental data**

Supplemental files 1,2,5

<https://drive.google.com/file/d/1oGEg5fykBSvM0sg0XMmMbEi1e7rA58SH/view?usp=drive_web>

Supplemental files 3,4

<https://drive.google.com/file/d/1LJFVduimnO0SUwyCDjpYKyoQ9OuoDSdw/view?usp=drive_web>
